# Highlighting the factors governing transglycosylation in the GH5_5 endo-1,4-β-glucanase RBcel1

**DOI:** 10.1107/S2059798321013541

**Published:** 2022-02-18

**Authors:** Laetitia Collet, Corinne Vander Wauven, Yamina Oudjama, Moreno Galleni, Raphaël Dutoit

**Affiliations:** a LABIRIS, 1 Avenue Emile Gryzon, 1070 Brussels, Belgium; b Center for Protein Engineering (CIP), Biological Macromolecules, University of Liège, 13 Allée du 6 Août, 4000 Liège, Belgium

**Keywords:** glycosyl hydrolase family 5, endo-1,4-β-glucanases, transglycosylation, RBcel1

## Abstract

Transglycosylating glycoside hydrolases (GHs) offer great potential for the enzymatic synthesis of oligosaccharides. Although knowledge is progressing, there is no unique strategy to improve the transglycosylation yield. Obtaining efficient enzymatic tools for glycan synthesis with GHs remains dependent on an improved understanding of the molecular factors governing the balance between hydrolysis and transglycosylation. This enzymatic and structural study of RBcel1, a transglycosylase from the GH5_5 subfamily isolated from an uncultured bacterium, aims to unravel such factors. The size of the acceptor and donor sugars was found to be critical since transglycosylation is efficient with oligosaccharides at least the size of cellotetraose as the donor and cellotriose as the acceptor. The reaction pH is important in driving the balance between hydrolysis and transglycosylation: hydrolysis is favored at pH values below 8, while transglycosylation becomes the major reaction at basic pH. Solving the structures of two RBcel1 variants, RBcel1_E135Q and RBcel1_Y201F, in complex with ligands has brought to light some of the molecular factors behind transglycosylation. The structure of RBcel1_E135Q in complex with cellotriose allowed a +3 subsite to be defined, in accordance with the requirement for cellotriose as a transglycosylation acceptor. The structure of RBcel1_Y201F has been obtained with several transglycosylation intermediates, providing crystallographic evidence of transglycosylation. The catalytic cleft is filled with (i) donors ranging from cellotriose to cellohexaose in the negative subsites and (ii) cellobiose and cellotriose in the positive subsites. Such a structure is particularly relevant since it is the first structure of a GH5 enzyme in complex with transglycosylation products that has been obtained with neither of the catalytic glutamate residues modified.

## Abstract 2

An enzymatic and structural study identifying the factors influencing the transglycosylase activity of RBcel1, a member of glycoside hydrolase family 5, is reported.

## 1. Introduction

Awareness of the role of glycans in biological processes has stimulated research and has led to a significant expansion in knowledge over the last two decades (Varki, 2017 ▸). Oligosaccharides and glycoconjugates are currently important therapeutic targets for many diseases. Their pure homogeneous forms are also increasingly used in glycobiology research and vaccine development (Boltje *et al.*, 2009 ▸). Their production, however, remains challenging (Geyer & Geyer, 2006 ▸; Boltje *et al.*, 2009 ▸; Kiessling & Splain, 2010 ▸; Wong & Krasnova, 2019 ▸). The use of enzymes for *in vitro* synthesis of oligosaccharides has been seen as a promising alternative to chemical synthesis in recent decades, mainly due to their high stereoselectivity and regioselectivity, and their action in mild aqueous conditions (Li & Wang, 2016 ▸; Benkoulouche *et al.*, 2019 ▸). Among them, transglycosylating glycoside hydrolases (GHs), known as transglycosylases, offer great potential for the synthesis of oligosaccharides as they do not require activated sugars as donors (Monsan *et al.*, 2010 ▸; Bissaro *et al.*, 2015 ▸; Danby & Withers, 2016 ▸).

Only a few characterized GHs from several families in the CAZy database have been described as transglycosylases. The overall mechanism of transglycosylation is well known and generally follows the same reaction pattern as the retaining mechanism of hydrolytic GHs (Crout & Vic, 1998 ▸; Bissaro *et al.*, 2015 ▸). Indeed, the capacity to transglycosylate is a direct consequence of the double-displacement mechanism. After the glycosylation step, the donor sugar is covalently bound to the enzyme within its negatively numbered subsites. Transglycosylation occurs when a sugar hydroxyl group is used as an acceptor instead of a water molecule during the deglycosylation step. The reaction yields, however, remain low due to simultaneous hydrolysis of the products (Abdul Manas *et al.*, 2018 ▸). It has been proposed that the ratio of hydrolysis to transglycosylation in transglycosylating GHs can be modulated by subtle molecular adjustments, such as modification of the donor/acceptor-binding sites and the orientation of the catalytic residues and the exclusion of water molecules from the catalytic site (Bissaro *et al.*, 2015 ▸; Abdul Manas *et al.*, 2018 ▸).

The occurrence of aromatic residues in the positive subsites of transglycosylating GHs is known to confer a high affinity for an acceptor sugar (Abdul Manas *et al.*, 2018 ▸). Notably, a Phe-clamp formed by two phenylalanine residues promotes interactions between the positive subsites and the acceptor sugar in the GH5_9 exo-1,3-β-glucanase from *Candida albicans* (Patrick *et al.*, 2010 ▸). Another adaptation in the positive subsites is an increased affinity for a sugar acceptor via a positive charge provided by an arginine residue, as in the +2 subsite of several fungal GH5_7 β-mannanases (Dilokpimol *et al.*, 2011 ▸; Rosengren *et al.*, 2012 ▸). An equivalent arginine residue has also been observed in the +2 subsites of two transglycosylating GH5_5 cellulases: RBcel1 and Ps_Cel5A (Delsaute *et al.*, 2013 ▸; Dutoit *et al.*, 2019 ▸). However, this adaptation has not been observed in other structurally characterized GH5_5 enzymes (Dutoit *et al.*, 2019 ▸). Therefore, the characterization of new transglycosylases is needed to identify the particularities and subtle differences in their catalytic clefts and to shed light on their genuine function.

RBcel1, an endoglucanase of the GH5_5 subfamily isolated from an uncultured bacterium, has aroused interest because of its ability to polymerize cellooligosaccharides *in vitro* under near-physiological conditions (Berlemont *et al.*, 2009 ▸). In a recent study, we reported that a single substitution in its active site, Tyr201Phe, stabilizes the formation of the glycosyl-enzyme covalent intermediate (GEI; Collet *et al.*, 2021 ▸). The structure of the GEI obtained with the natural oligosaccharide cellotriose delivered a series of snapshots of the reaction mechanism. Tyr201 is a highly conserved residue among GH5 family members and is believed to play an important role in both glycosylation and deglycosylation (Ducros *et al.*, 1995 ▸; Sakon *et al.*, 1996 ▸; Collet *et al.*, 2021 ▸; Gonçalves *et al.*, 2012 ▸; Kim & Ishikawa, 2011 ▸; Zheng *et al.*, 2012 ▸). The Tyr201Phe variant of RBcel1 retains transglycosylation activity, although it is reduced. Obtaining its structure with transglycosylation products could help to identify the structural motifs and residues involved in cellooligosaccharide synthesis.

Here, we report the influence of various factors on the transglycosylation activity of RBcel1. Firstly, the size of the acceptor sugar was found to be decisive and must be of at least three sugar units. This implies the existence of a third positive subsite which had not previously been described (Delsaute *et al.*, 2013 ▸; Collet *et al.*, 2021 ▸). The structure of the Glu135Gln variant of RBcel1 was solved in complex with cellotriose. It allowed the identification of the residues defining the +3 subsite. Secondly, in RBcel1 the reaction pH had a strong effect on hydrolysis and on the accumulation of transglycosylation products. Notably, at a pH above 8 hydrolysis is reduced and transglycosylation products tend to accumulate. Thirdly, the structure of the Tyr201Phe variant of RBcel1 was obtained in complex with transglycosylation products, highlighting several new key features of transglycosylation.

## 2. Materials and methods

### 2.1. Cloning and mutagenesis of RBcel1

The original pET-22b-RBcel1 expression vector used for the production of wild-type RBcel1 protein (RBcel1_WT) has been described previously (Berlemont *et al.*, 2009 ▸), as well as the pBAD-RBcel1 vector for the production of the RBcel1 variant with Tyr201 substituted with a phenylalanine residue (RBcel1_Y201F; Collet *et al.*, 2021 ▸). The RBcel1 variant with Glu135 substituted with a glutamine residue (RBcel1_E135Q) was produced from pET-22b-RBcel1 after targeted muta­genesis with the QuikChange Site-Directed Mutagenesis Kit (Agilent). All genetic constructs were verified by sequencing (Genetic Service Facility, University of Antwerp). *Escherichia coli* strain MC1061 was used for cloning and *E. coli* strain BL21 (DE3) was used for heterologous expression.

### 2.2. Production and purification of RBcel1

All constructs contain the original RBcel1 signal sequence which allows export of the recombinant protein into the periplasm. Consequently, all RBcel1 variant proteins were purified from periplasmic extracts. Cells producing RBcel1_WT and RBcel1_E135Q were grown and induced as described previously (Berlemont *et al.*, 2009 ▸). RBcel1_Y201F was produced as described previously (Collet *et al.*, 2021 ▸). Briefly, periplasmic extracts prepared as described in Garsoux *et al.* (2004 ▸) were loaded onto an ion-exchange column (SOURCE 15Q, 12 ml, GE Healthcare) equilibrated in 20 m*M* Tris–HCl pH 8.5. Proteins were eluted using a linear NaCl gradient from 0 to 500 m*M*. The fractions containing RBcel1 were pooled, concentrated and loaded onto a size-exclusion chroma­tography column (Superdex 75, 120 ml, GE Healthcare) equilibrated with 20 m*M* sodium phosphate buffer pH 6.5. The relevant fractions were pooled and concentrated using an Amicon ultrafiltration unit (Merck Millipore) with a 10 kDa cutoff. The purity was checked by SDS–PAGE. Protein concentrations were calculated using theoretical extinction coefficients (Δɛ_280_ = 80 455 *M* ^−1^ cm^−1^ for RBcel1_WT and RBcel1_E135Q and 78 965 *M* ^−1^ cm^−1^ for RBcel1_Y201F).

### 2.3. Enzyme-activity assays

#### 2.3.1. Determination of kinetic parameters

The hydrolytic activity was assayed using 4-nitrophenyl β-glucoside (PNP β-G1), 2-chloro-4-nitrophenyl β-cellobioside (ClPNP β-G2), 2-chloro-4-nitrophenyl β-cellotrioside (ClPNP β-G3), 2-chloro-4-nitrophenyl β-cellotetraoside (ClPNP β-G4) and 2-chloro-4-nitrophenyl β-cellopentaoside (ClPNP β-G5) as substrates. ClPNP derivatives and PNP β-G1 were purchased from Megazyme and Sigma, respectively. The release of 4-nitrophenol (PNP) and 2-chloro-4-nitrophenol (ClPNP) was monitored by measuring the absorbance at 400 nm over 2 min. The amount of ClPNP released is directly proportional to the rate of substrate hydrolysis. Kinetic parameters were determined under the initial rate conditions by nonlinear regression of the Michaelis–Menten equation. 0.2 µ*M* enzyme was incubated with substrate in the range 0.3–6 m*M*. The reaction was conducted at 37°C in 20 m*M* sodium phosphate buffer pH 6.5. The kinetic parameters of RBcel1_E135Q and RBcel1_Y201F were determined using ClPNP β-G1 as a substrate in the range 0.125–6 m*M*. The enzyme concentration was adapted due to the impaired activity of the variants: 36 µ*M* for RBcel1_E135Q and 22 µ*M* for RBcel1_Y201F.

#### 2.3.2. Thin-layer chromatography

Thin-layer chromatography (TLC) was used to visualize the reaction products resulting from the activity of RBcel1 on different ClPNP β-cellooligosaccharides. Unless stated otherwise, the reaction mixtures consisted of 2 m*M* ClPNP β-cellooligosaccharide in 20 m*M* sodium phosphate buffer pH 6.5. 10 µ*M* enzyme was added to the reaction mixture at 4°C and then incubated for 5 min at 25°C. The effect of pH on activity was determined by incubating 10 µ*M* enzyme with 2 m*M* ClPNP β-G3 for 5 min at 25°C in 20 m*M* citrate–phosphate–CHES buffer with the pH adjusted in the range pH 4 to pH 9 (Berlemont *et al.*, 2009 ▸). Size-reference ladders were prepared by mixing PNP β-G1, ClPNP β-G2, ClPNP β-G3, glucose (G1), cellobiose (G2), cellotriose (G3), cellotetraose (G4), cellopentaose (G5) and cellohexaose (G6) at a final concentration of 1 m*M* each. G2 to G6 cellooligosaccharides were purchased from Megazyme. The reaction was stopped by heating the samples at 95°C for 5 min. After centrifugation at 10 000*g* for 2 min, 40 µl of the reaction mixture was evaporated to ∼8 µl, which was spotted onto silica gel 60 TLC glass plates (Merck Millipore). Chromatograms were developed in a mixture of butanol/acetic acid/water [50:25:25(*v*:*v*:*v*)]. Reaction products were revealed by spraying the plates with 1-naphthol (2%) in ethanol/concentrated sulfuric acid/water [83/10/75(*v*:*v*:*v*)] and heating at 121°C for 10 min.

#### 2.3.3. PACE analysis

Polyacrylamide carbohydrate electrophoresis (PACE) was performed as described previously (Collet *et al.*, 2021 ▸) with the following adaptations. To determine the potential acceptors used in transglycosylation, 4,6-*O*-benzylidene-2-chloro-4-nitrophenyl-β-cellotrioside (ClPNP β-BG3; from the K-CellG3 cellulase assay kit, Megazyme) was used as a donor. G1, G2, G3 and G4 were used as donor sugars. Unless stated otherwise, 1 m*M* ClPNP β-BG3 was mixed with 1 m*M* of the acceptor sugar in 20 m*M* sodium phosphate buffer pH 6.5. As ClPNP β-BG3 is supplied as a solution in DMSO, the reaction mixture contained 10%(*v*/*v*) DMSO. The reaction was started by adding 10 µ*M* enzyme to the reaction mixture kept at 4°C and was immediately incubated at 25°C for 10 min. The reaction was stopped by adding 0.5%(*v*/*v*) formic acid. To separate 4,6-*O*-benzylidene-β-­cellotrioside (BG3) from G4, the gel was run with a constant current of 18 mA for 90 min instead of 60 min. The hydrolysis and transglycosylation products of RBcel1_Y201F were also determined by PACE using G3 under conditions close to those of crystallogenesis. 166 µ*M* RBcel1_Y201F was incubated with 10 m*M* G3 at 20°C in 0.1 *M* Tris pH 7. Samples were taken at different incubation times (1 min to 20 days) and the reaction was stopped by adding 0.5%(*v*/*v*) formic acid prior to PACE.

### 2.4. Crystallization

RBcel1_E135Q and RBcel1_Y201F (Table 1 ▸) were crystallized using the hanging-drop vapor-diffusion method at 293 K. Crystallization was set up in EasyXtal plates (Qiagen). RBcel1_Y201F, stored at a concentration of 400 µ*M* in 20 m*M* sodium phosphate pH 6.5, was mixed in a 1:1 ratio with a well buffer consisting of 0.1 *M* Tris, 17.5% PEG 600 pH 7. For co-crystallization of RBcel1_Y201F with cellotriose (G3), the enzyme was incubated with 1 m*M* G3 for 1 h at 4°C. Drops consisted of 2 µl of the enzyme–G3 reaction mixture and 2 µl 0.1 *M* Tris, 20.5% PEG 600 pH 7.0. Single crystals appeared after a few hours and grew to maximum dimensions within two days at 293 K. Crystals were picked for cryogenization after one week. RBcel1_E135Q was crystallized by mixing 2 µl 385 µ*M* protein in 20 m*M* sodium phosphate buffer pH 6.5 with 2 µl 0.1 *M* Tris–HCl, 17.5% PEG 600 pH 7.4. To co-crystallize RBcel1_E135Q with G3, 1 m*M* G3 was added to a drop consisting of 2 µl 385 µ*M* protein solution and 2 µl 0.1 *M* Tris–HCl, 17.5% PEG 600 pH 7.4. Microseeding was necessary to improve the crystal shape and size. Before picking up the crystals, the drops were equilibrated for 2 h against a 500 µl reservoir consisting of 0.1 *M* Tris, 30% PEG 600 pH 7.4 for cryoprotection. The crystallization conditions are summarized in Table 2 ▸.

### 2.5. Data collection and processing

Diffraction data for RBcel1_E135Q were collected on the FIP-BM30A beamline (Ferrer, 2001 ▸) at ESRF, Grenoble, France and those for RBcel1_E135Q in complex with G3 and RBcel1_Y201F on the PROXIMA-2 beamline at SOLEIL, Saint-Aubin, France. Diffraction data were indexed using the *XDS* program package (Kabsch, 2010 ▸). The statistics of data collection and indexing are summarized in Table 3 ▸. The four structures were determined by molecular replacement with *Phaser-MR* in *Phenix* (McCoy *et al.*, 2007 ▸; Liebschner *et al.*, 2019 ▸) using the coordinates of RBcel1 (PDB entry 4ee9; Delsaute *et al.*, 2013 ▸) as a search model. The models were built using *phenix.autobuild* (Liebschner *et al.*, 2019 ▸) and *Crystallographic Object-Oriented Toolkit* (*Coot*; Emsley *et al.*, 2010 ▸). Multiple rounds of refinement were performed using *phenix.refine* (Liebschner *et al.*, 2019 ▸). The stereochemical quality of the models was assessed using *MolProbity* (Chen *et al.*, 2010 ▸). The structure solutions and refinement statistics for the four structures are presented in Table 4 ▸. Protein–ligand interactions were analyzed using *PDBeMotif* (Golovin & Henrick, 2008 ▸). Structures were illustrated using the *PyMOL* molecular-graphics system version 0.9 (Schrödinger).

## 3. Results and discussion

### 3.1. Importance of the size of the donor sugars

Previously, the catalytic cleft of RBcel1 has been described as consisting of four negatively and two positively numbered subsites. The six subsites were identified by (i) comparing the structure of RBcel1 with those of structural homologs belonging to the GH5 family (Delsaute *et al.*, 2013 ▸) and (ii) obtaining the structure of the Glu135Ala mutant in complex with G3 (Collet *et al.*, 2021 ▸). However, it remained unclear whether the subsites had to be completely occupied for hydrolysis or transglycosylation to occur. During the first step of the reaction, the part of the substrate located in the negatively numbered subsites (following the nomenclature established by Davies *et al.*, 1997 ▸) becomes covalently linked to the nucleophilic residue, while the part initially hosted in the positive subsites is released. Chromogenic cellooligosaccharides have been used to determine the effect of substrate size on the first displacement (Desmet *et al.*, 2007 ▸).

The activity of RBcel1 was assayed on chromogenic cellooligosaccharides of increasing size and the kinetic parameters were determined. As shown in Table 5 ▸, no significant activity was observed on PNP β-G1 or ClPNP β-G2, in contrast to longer substrates, indicating that at least three negatively numbered subsites must be occupied for hydrolysis to occur. RBcel1 displayed the highest catalytic efficiency with ClPNP β-G4 as a substrate, which was nearly ten times that with ClPNP β-G3. Such a difference in catalytic efficiency is mainly due to the affinity for the substrate. Indeed, the *K*_m_ for ClPNP β-G4 was 0.3 µ*M*, compared with 2.4 µ*M* for ClPNP β-G3, while both substrates were hydrolysed at a similar rate. Since the catalytic efficiency was not increased with ClPNP β-G5, only four negatively numbered subsites of RBcel1 need to be occupied, which is consistent with the structural analysis of the catalytic cleft.

The reaction products resulting from hydrolysis of the chromogenic cellooligosaccharides were analyzed by TLC. As expected, no activity was observed with PNP β-G1 and ClPNP β-G2 (Supplementary Fig. S1). On the other hand, RBcel1 effectively degraded ClPNP β-G3 and ClPNP β-G4 into various products, including transglycosylation products. For instance, RBcel1 generated the hydrolysis products G2 and G3 from ClPNP β-G3 and the transglycosylation products G4 and G5 (Supplementary Fig. S1). ClPNP β-G1 and ClPNP β-G2 were also formed during the degradation of ClPNP β-G3. Considering these results, the products are more likely to be produced by the hydrolysis of transglycosylation products rather than from an alternate hydrolysis of the substrate. Consequently, the kinetic parameters shown in Table 5 ▸ are probably underestimated since both hydrolysis and transglycosylation occur. For further characterization of the transglycosylase activity, ClPNP β-G3 was preferred over ClPNP β-G4, which contained several contaminants that hinder the interpretation of the data (Supplementary Fig. S1).

### 3.2. Impact of the pH on transglycosylation

**Figure 1:**
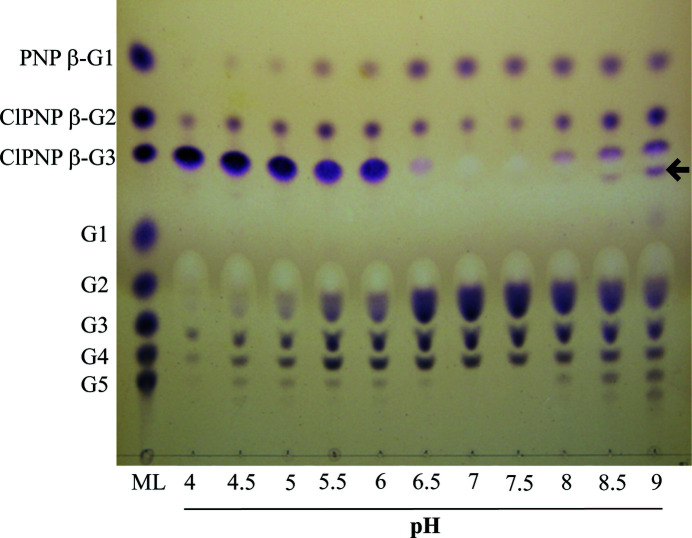
TLC analysis of the products of RBcel1_WT activity on ClPNP β-G3 over a range of pH values from 4 to 9. 10 µ*M* enzyme was incubated with 2 m*M* ClPNP β-G3 in 20 m*M* citrate–phosphate–CHES buffer for 5 min at 25°C. The following cellooligosaccharides and ClPNP derivatives were used as standards (1 m*M* of each; lane ML): glucose (G1), cellobiose (G2), cellotriose (G3), cellotetraose (G4), cellopentaose (G5), 4-­nitrophenyl β-glucoside (PNP β-G1), 2-chloro-4-nitrophenyl β-cellobioside (ClPNP β-G2) and 2-chloro-4-nitrophenyl β-cellotrioside (ClPNP β-­G3). The presence of 2-chloro-4-nitrophenyl β-cellotetraoside is marked by an arrow.

In their review, Abdul Manas *et al.* (2018 ▸) discuss different factors that favor either hydrolysis or transglycosylation. Among them, pH can influence transglycosylation by modulating the protonation states of catalytically important residues. Consequently, the influence of the pH on the ability of RBcel1 to transglycosylate was investigated using ClPNP β-G3. RBcel1 was incubated with ClPNP β-G3 at different pH values and the reaction products were analyzed by TLC. As shown in Fig. 1 ▸, ClPNP β-G3 was almost completely consumed at pH 6.5 to 7.5, which correspond to the previously published optimum pH of the hydrolysis activity (Berlemont *et al.*, 2009 ▸). G4 was observed at all pH values, indicating that transglycosylation occurred over a wide range of pH values. The transglycosylation products, however, varied significantly. At the optimum pH for hydrolysis, ranging from pH 6.5 to 7.5, their diversity was the lowest, probably due to rapid hydrolysis of the newly formed transglycosylation products. At these pH values the spot intensities of the hydrolysis products (*i.e.* G2, G3, ClPNP β-G2 and ClPNP β-G1) were accordingly the highest.

At pH values below 6.5 the degradation rate of ClPNP β-G3 was slower, enabling the detection of products formed at the very beginning of the reaction. For instance, G4 and ClPNP β-G2 are the only products observed at pH 4 (see Fig. 1 ▸), and probably result from hydrolysis of the transglycosylation product ClPNP β-G6. Above pH 8 the diversity of transglycosylation products was the highest, with a bountiful accumulation of G4, G5, G6 and even ClPNP β-G4. Such an accumulation could not be explained by a slower reaction rate since the initial substrate (ClPNP β-G3) was almost completely consumed.

To our knowledge, the influence of pH on transglycosylation remains ill-described due to a scarcity of data. For instance, Lundemo and coworkers reported that pH influences hydrolysis only, without any effect on transglycosylation, for a GH1 β-glucosidase from *Thermotoga neapolitana* (Lundemo *et al.*, 2017 ▸). On the contrary, Oikawa and coworkers observed that the transglycosylation activity was increased at acidic pH for an endo-β-glucanase from *Rhodotorula glutinis* (Oikawa *et al.*, 2001 ▸). A likely explanation for the accumulation of transglycosylation products at high pH values could reside in the protonation state of Glu135, the catalytic acid/base residue (Glu_A/B_) in RBcel1. We propose that at basic pH values Glu135 is maintained in a deprotonated state. As previously described for the RBcel1_E135A variant (Collet *et al.*, 2021 ▸), a lack of protonic assistance from Glu135 would totally prevent the hydrolysis of natural sugars but would still provide assistance as a base essential to transglycosylation. Consequently, higher pH values promote transglycosylation with ClPNP β-G3 and prevent hydrolysis of the newly formed products.

### 3.3. Importance of the size of the acceptor sugar for the transglycosylation activity

As mentioned earlier, RBcel1 can produce transglycosylation products with ClPNP β-G3. Transglycosylation occurs when an acceptor sugar occupies the positively numbered subsites. The acceptor sugar is activated by Glu_A/B_ and releases the GEI during the second step of the reaction. Although two positively numbered subsites have been described in the structure of RBcel1 (Delsaute *et al.*, 2013 ▸; Collet *et al.*, 2021 ▸), it was still unknown whether the size of the acceptor sugar influences transglycosylation. This aspect was investigated here using ClPNP β-BG3 as a donor and various cellooligosaccharides, from G1 to G4, as acceptors. ClPNP β-BG3 is a chromogenic G3 with its nonreducing end protected by a benzylidene group. Therefore, it cannot be used as an acceptor. As TLC could not be performed with benzylidene-linked oligosaccharides, PACE was used to analyze the reaction products. Inherently to this method, however, the chromogenic derivatives cannot be observed due to the absence of a reducing end.

**Figure 2:**
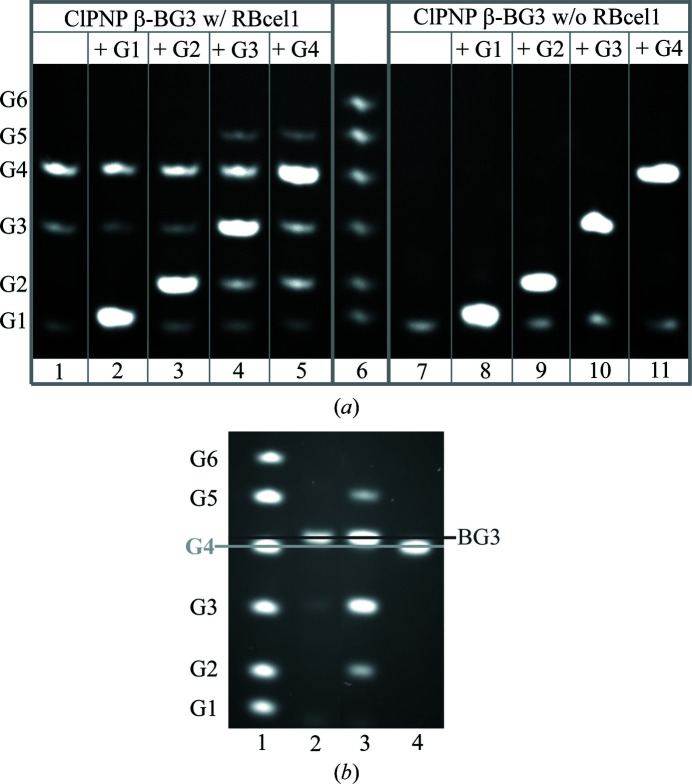
(*a*) PACE analysis of the activity of RBcel1_WT on 4,6-*O*-benzylidene-2-chloro-4-nitrophenyl-β-cellotrioside (ClPNP β-BG3) with different sugar acceptors. Lanes 1–5 show the reaction products when 1 m*M* ClPNP β-­BG3 was incubated with 10 µ*M* enzyme for 10 min at 25°C: lane 1, without an acceptor; lane 2, with 1 m*M* G1; lane 3, with 1 m*M* G2; lane 4, with 1 m*M* G3; lane 5, with 1 m*M* G4. Lanes 7–11 show the corresponding control reaction mixtures incubated without enzyme: lane 7, ClPNP β-­BG3; lane 8, ClPNP β-BG3 + G1; lane 9, ClPNP β-BG3 + G2; lane 10, ClPNP β-BG3 + G3; lane 11, ClPNP β-BG3 + G4. Lane 6: oligosaccharide size ladder comprising G1, G2, G3, G4, G5 and G6. (*b*) PACE analysis to validate 4,6-*O*-benzylidene-β-cellotrioside (BG3) as a product of the hydrolysis of ClPNP β-BG3. Lane 1, oligosaccharide size ladder comprising G1, G2, G3, G4, G5 and G6; lane 2, 1 m*M* BG3 incubated without enzyme; lane 3, products of the incubation of 1 m*M* ClPNP β-­BG3 with 1 m*M* G3 and 10 µ*M* enzyme; lane 4, 1 m*M* G4 incubated without enzyme.

As shown in Fig. 2 ▸(*a*), in the absence of a cellooligosaccharide acceptor the hydrolysis of ClPNP β-BG3 produced two products with the apparent sizes of G4 and G3. Since the nonreducing end of the substrate is blocked, the former should therefore correspond to 4,6-*O*-benzylidene-β-d-cellotriose (BG3) and not to G4, and the latter to BG2 and not to G3. Indeed, when PACE was performed with a longer migration time, BG3 clearly separated from G4 (Fig. 2 ▸ *b*). Unequivocally, the hydrolysis of ClPNP β-BG3 generated BG3, and the use of this substrate as a donor was validated. In the presence of G1 as an acceptor only the BG3 hydrolysis products were observed, meaning that no transglycosylation had occurred (Fig. 2 ▸ *a*). With G2 as an acceptor, a faint band corresponding to G6 was observed, indicating that transglycosylation had occurred (Fig. 2 ▸ *a*). With G3 and G4 as acceptors, a transglycosylation product corresponding to G5 accumulated in the reaction mixture along with G2 (Fig. 2 ▸ *a*). Our results suggest that transglycosylation is dependent on the length of the acceptor sugar and needs an acceptor of at least three glucose units to proceed efficiently.

### 3.4. Positioning of G3 as the acceptor sugar: structure of RBcel1_E135Q with G3

G3 being a better acceptor than G2 is an indication of the existence of a third positively numbered subsite in the catalytic cleft of RBcel1. This additional subsite, however, had not been observed in the previous characterization of the RBcel1 structure. In the structure of RBcel1_E135A in complex with G3 (Collet *et al.*, 2021 ▸), a G3 molecule was found spanning the −1 to +2 subsites, and no G3 was found solely occupying positively numbered subsites. In this variant, the position of G3 could have resulted from the lack of steric constraints. Thus, to mimic the presence of the Glu_A/B_ residue, the variant RBcel1_E135Q was generated to determine its structure with G3. The activity of this variant was dramatically impaired with ClPNP β-G3 as substrate, with a *k*_cat_ of 0.219 ± 0.009 × 10^−3^ min^−1^ and a *K*_m_ of 0.263 ± 0.041 m*M*. The kinetic parameters strongly indicate that both the glycosylation and deglycosylation steps are affected by the substitution of Glu135 with a glutamine residue. RBcel1_E135Q was then co-crystallized with G3 and a data set was obtained, and the structure of the complex was solved at 1.73 Å resolution.

**Figure 3:**
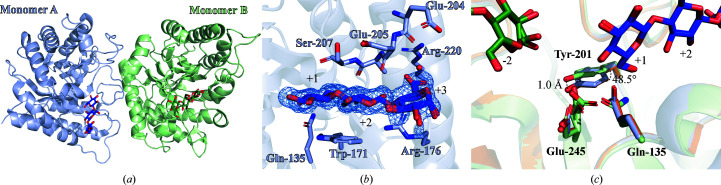
(*a*) Overall structure of RBcel1_E135Q in complex with G3 (PDB entry 7p6h). Monomer *A* and monomer *B* are represented in light blue and light green, respectively, while their bound G3 molecules are colored dark blue and dark green, respectively. (*b*) Close-up view of the +1 to +3 subsites consisting of Trp171, Arg176, Glu204, Glu205, Ala206 and Ser207. The water molecule interacting with the glucose unit in the +3 subsite is represented as a red sphere. The 2*F*_o_ − *F*_c_ map around G3 is shown as a blue mesh. (*c*) Structural alignment of RBcel1_E135Q in complex with G3 (in blue and green for monomer *A* and monomer *B*, respectively) with the apo form of RBcel1_E135Q (PDB entry 7p6g, orange). The positions of three residues of the −1 subsite are shown: Gln135 (Glu_A/B_ substituted by a glutamine residue), Tyr201 and Glu245 (the catalytic nucleophile).

The asymmetric unit contains two monomers, each in complex with a differently positioned G3 molecule (Fig. 3 ▸ *a*). In monomer *B* the G3 molecule occupies the −4 to −2 subsites (Supplementary Fig. S2), but in monomer *A* it occupies the positively numbered subsites only, lying beyond the +2 subsite (Fig. 3 ▸ *b*). Its third glucose unit is placed in a poorly defined +3 subsite, making a few interactions with the carbonyl of Asp205 and a water molecule coordinated by the carbonyl of Glu204 and the side chain of Arg226 (Fig. 3 ▸ *b*). The catalytic residues are placed differently depending on where the ligand is positioned. The side chain of the nucleophile Glu245 is positioned as described for RBcel1_WT in monomer *A* and the apo form of RBcel1_E135Q. In monomer *B* it adopts several conformations: its carboxylate is rotated by 50° along the C^β^ axis, leading to a rotamer change from **mt**-10° to **tt**0° (Fig. 3 ▸ *c*). The same rotation of Glu245 was previously described during the formation of the GEI (Collet *et al.*, 2021 ▸). It seems that occupancy of the farther negative subsites is required for Glu245 to be correctly positioned to form the GEI. The **mt**-10° to **tt**0° rotation seems to impact the position of the side chain of Tyr201. When Glu245 adopts the **tt**0° conformation, as seen in monomer *B* (Fig. 3 ▸ *c*), the hydroxyl group of Tyr201 is displaced by 1 Å compared with monomer *A* and the apo form of RBcel1_E135Q. The aromatic plane of Tyr201 is also tilted by 48.5° in monomer *B*. Furthermore, OE1 of Gln135 is rotated by 50° in monomer *B* compared with the position of OE1 of Gln135 in monomer *A* and the apo form of RBcel1_E135Q (Fig. 3 ▸ *c*). To our knowledge, such a displacement of the acid/base residue has not been reported for other GH5 enzymes.

### 3.5. Snapshots of RBcel1_Y201F on the way to transglycosylation

**Figure 4:**
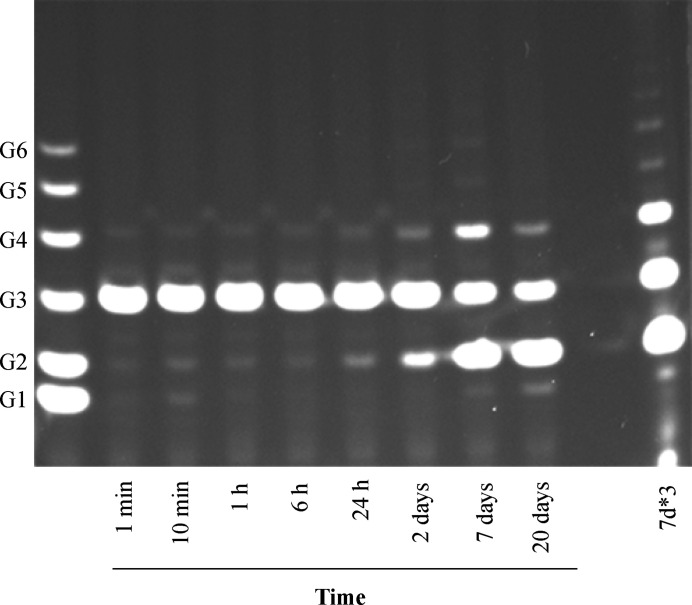
PACE analysis of RBcel1_Y201F activity on G3 after different incubation times. 166 µ*M* RBcel1_Y201F was incubated with 10 m*M* G3 at 20°C in 0.1 *M* Tris buffer pH 7. 7d*3 corresponds to the reaction incubated for seven days loaded three times.

Our previous study of RBcel1 showed that the substitution of Tyr201 with a phenylalanine residue drastically slows the deglycosylation step, resulting in accumulation of the GEI (Collet *et al.*, 2021 ▸). The GEI, however, was released over time and the transglycosylase activity of RBcel1_Y201F was maintained. Therefore, it should be possible to obtain snapshots of the transglycosylation from the structure of this variant in complex with cellooligosaccharides. The kinetic parameters of RBcel1_Y201F were determined using ClPNP β-G3. The *k*_cat_ and *K*_m_ were 0.194 ± 0.023 × 10^−3^ min^−1^ and 0.077 ± 0.010 m*M*, respectively, confirming that Tyr201 is important in catalysis. The activity of RBcel1_Y201F was also measured with G3 at an enzyme:substrate ratio close to the crystallogenesis conditions. As shown in Fig. 4 ▸, after seven days of incubation transglycosylation products were observed such as G4 (the main transglycosylation product), G5 and G6. Consequently, RBcel1_Y201F was co-crystallized with G3 for one week prior to cryogenization and data collection. Its structure was solved at 1.74 Å resolution along with the structure of the apo form for comparison.

**Figure 5:**
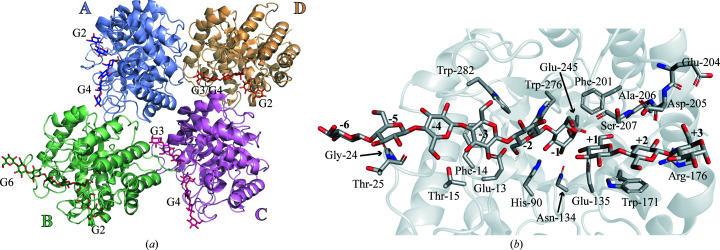
(*a*) Overall structure of RBcel1_Y201F co-crystallized with cellotriose (PDB entry 7p6j). The monomers in the asymmetric unit are highlighted as follows: monomer *A* in blue, monomer *B* in green, monomer *C* in pink and monomer *D* in orange. Their respective ligands are shown in darker colors. (*b*) Representation of the whole catalytic cleft of RBcel1 consisting of the −6 to −1 subsites and the +1 to +3 subsites. The residues interacting with the ligand are emphasized.

**Figure 6:**
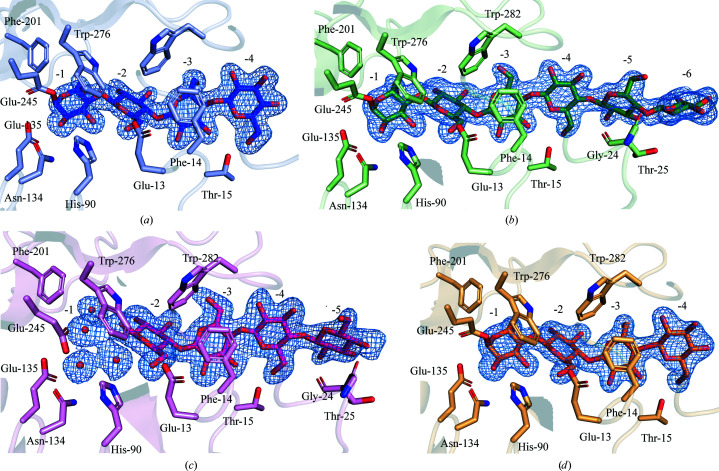
Close-up view of the donor-binding sites in the structure of RBcel1_Y201F co-crystallized with G3. (*a*) Monomer *A* with G4 in its −1 to −4 subsites. (*b*) Monomer *B* with G6 in its −1 to −6 subsites. (*c*) Monomer *C* with G4 in its −2 to −5 subsites and four water molecules in its −1 subsite. (*d*) Monomer *D* with G3 and G4 in its −1 to −4 subsites. The residues composing each subsite are emphasized. The 2*F*_o_ − *F*_c_ map around each cellooligosaccharide is shown as a blue mesh.

**Figure 7:**
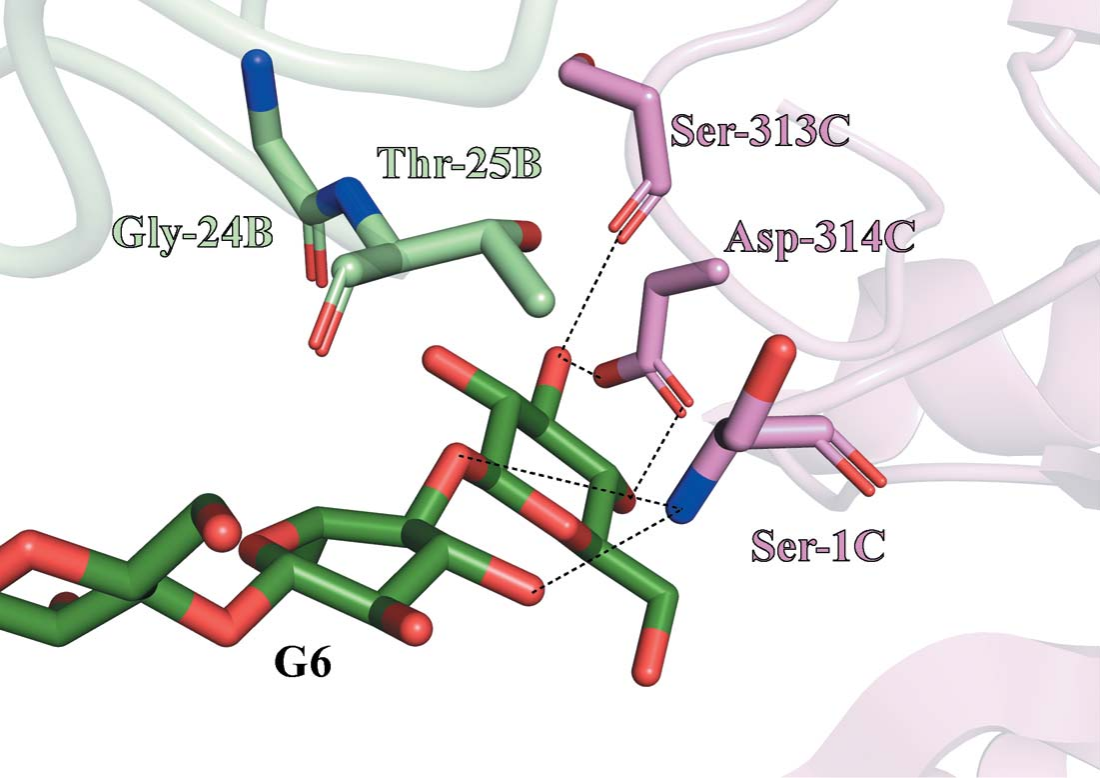
Close-up view of the −5 and −6 subsites of monomer *B* in the structure of RBcel1_Y201F in complex with G3. Monomer *B* and its bound G6 are shown in green. Gly24 and Thr25 composing the −5 and −6 subsites are emphasized. Additional interactions with G6 are provided from monomer *C* of a neighboring asymmetric unit (shown in pink). Ser1, Ser313 and Asp314 of monomer *C* are emphasized as well as their interactions with G6 (dashed lines).

The structure of the apo form of RBcel1_Y201F contains one monomer per asymmetric unit. A Tris molecule resides in the −1 subsite, as in the structure of RBcel1_WT. The structure of RBcel1_Y201F co-crystallized with G3 contains four monomers per asymmetric unit, with each monomer containing ligands ranging from G2 to G6 in the catalytic cleft (Fig. 5 ▸ *a*). The presence of cellooligosaccharides longer than G3 within the negatively numbered subsites provides crystallographic evidence for transglycosylation. The whole catalytic cleft is summarized in Fig. 5 ▸(*b*), emphasizing the residues interacting with the ligands. The real-space correlation coefficients of each ligand are presented in Supplementary Table S1. In monomer *A*, a G4 molecule covalently bound to Glu245 occupies the −4 to −1 subsites (Fig. 6 ▸ *a*). In monomer *D*, G3 and G4 were placed in the negative subsites according to real-space correlation coefficients and median *B* factors (Supplementary Table S1), with occupancies of 0.13 and 0.83 for G3 and G4, respectively. They are both covalently bound to Glu245 (Fig. 6 ▸ *d*). Monomer *C* has G2, G3 and G4 molecules placed in the −5 to −2 subsites (*i.e.* not covalently bound to Glu245). Intriguingly, electron density was also observed at the −1 subsite which cannot be explained by a sugar ring (Fig. 6 ▸ *c*). Several water molecules were placed in the model, but they are not sufficient to account for this excess of density. Unexpectedly, a G6 molecule is covalently bound to Glu245 in monomer *B* (Fig. 6 ▸ *b*). Only four negatively numbered subsites have been described so far (Delsaute *et al.*, 2013 ▸; Collet *et al.*, 2021 ▸). However, the presence of G6 in the negatively numbered subsites of RBcel1 in monomer *B* allowed the definition of two additional subsites: −5 and −6. Inside these, the sugar moieties interact with the carbonyl of Gly24 and Thr25 (Fig. *6b*). Additional interactions are provided from monomer *C* of a neighboring asymmetric unit (Fig. 7 ▸). Indeed, the N-terminal amine of Ser1 interacts with O3 and O1 of the sugar moieties in the −5 and −6 subsites, respectively. In addition, the carbonyl of Ser313 and the carboxylate of Asp314 closely interact with O3 and O4 at the nonreducing end of G6. Such interactions between monomer *C* and the covalently bound G6, however, could be fortuitous, resulting from a symmetry contact artifact.

**Figure 8:**
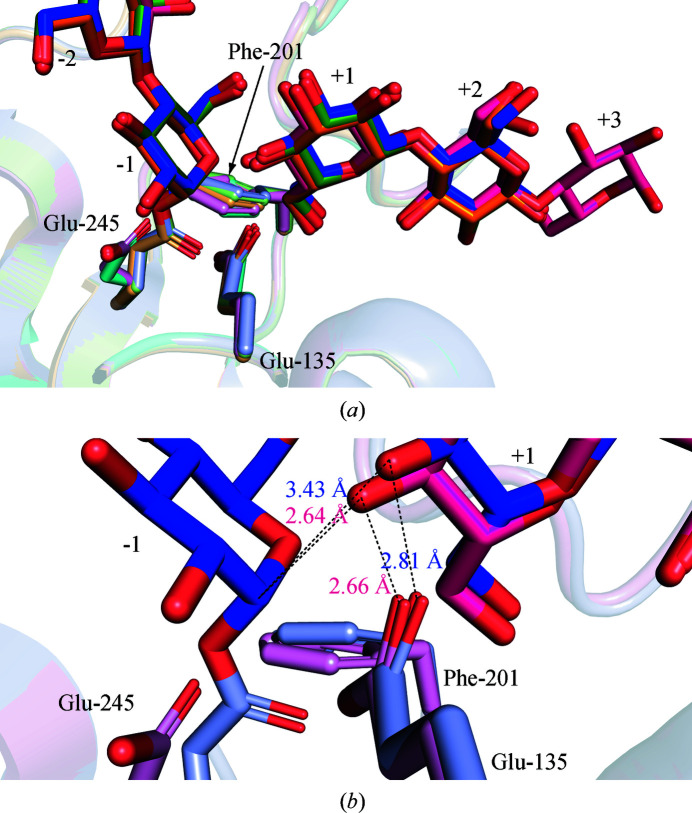
(*a*) Structural alignment of the four monomers of RBcel1_Y201F showing the position of the side chain of Glu245 and the ligands in the +1 to +3 subsites. Monomers *A*, *B*, *C* and *D* are represented in blue, green, pink and orange, respectively. To highlight the displacement of the side chain of Glu245 on the formation of the GEI, the apo-form structure of RBcel1_Y201F was added to the alignment (cyan; PDB entry 7p6i). G2 is found in the +1 to +2 subsites in monomers *A*, *B* and *D*, while G3 lies in the +1 to +3 subsites in monomer *C*. (*b*) Close-up view of the −1 and +1 subsites showing the structural changes when G3 is bound in the positively numbered subsites instead of G2. For the sake of clarity, only the superposition of monomer *A* (blue) with monomer *C* (pink) is shown. The distances between O4 of the acceptor sugar and C1 of the GEI or OE1 of Glu135 are given in the respective colors of monomers *A* and *C*.

As discussed in our previous study, a displacement of the Glu245 side chain to form the GEI, adopting the **tt**0° conformation instead of **mt**-10°, results in a change of electronic environment (Collet *et al.*, 2021 ▸). In the structure of RBcel1_Y201F in complex with G3, the same displacement of the side chain of Glu245 was observed in monomers *A*, *B* and *D*, where a ligand is covalently bound (Fig. 8 ▸ *a*). The Glu245 side chain, however, remains in the **mt**-10° conformation in monomer *C*, where G4 does not occupy the −1 subsite (Fig. 8 ▸ *a*). Since both conformations of the nucleophile residue occur in the same structure, the displacement must be related to the formation of the GEI and is not an artifact resulting from the Tyr201Phe substitution.

Regarding the positively numbered subsites, monomers *A*, *B* and *D* have a G2 molecule located in the +1 and +2 subsites (Fig. 5 ▸ and Supplementary Fig. S3). In monomer *C*, however, a G3 molecule occupies the +1 to +3 subsites, as in the structure of RBcel1_E135Q. According to the PACE analysis, RBcel1 uses both G2 and G3 as acceptors, although the latter is a better acceptor than the former. A superposition of the four monomers of the RBcel1_Y201F structure should allow a visualization of whether G2 and G3 adopt different positions (Fig. 8 ▸ *a*). The position of the G2 molecule was found to be nearly identical in monomers *A*, *B* and *D*. On the contrary, the occupancy of the third positive subsite clearly brings the glucose unit in the +1 subsite closer to Glu_A/B_. Indeed, G3 in monomer *C* is translated by 0.74 Å compared with G2, as shown in Fig. 8 ▸(*b*). As a result, the nonreducing end lies 2.66 Å from OE1 of Glu135 and, potentially, 2.64 Å from the reducing end of a bound donor. By comparison, these distances are increased to 2.81 and 3.43 Å, respectively, when G2 occupies the +1 and +2 subsites.

## 4. Conclusion

In this work, we have combined structural biology with enzymatic assays in order to better understand the molecular factors that govern transglycosylation in RBcel1. Substituting the conserved Tyr201 residue allowed us to resolve the first structure of a GH5 enzyme in complex with transglycosylation products with the two catalytic glutamate residues unmodified. By co-crystallizing RBcel1_Y201F with G3, the GEI was trapped with G3, G4 and G6 covalently bound to Glu245. Thanks to the presence of G6, the −5 and −6 subsites were identified, which could not be observed or predicted from previously released structures of RBcel1 (Delsaute *et al.*, 2013 ▸; Collet *et al.*, 2021 ▸). We have also shown that transglycosylation is dependent on the size of the donor and acceptor sugars. Transglycosylation products accumulate with an acceptor of at least three glucose units. Thus, for transglycosylation to proceed efficiently, a third positive subsite identified in the structure of Rbcel1_E135Q in complex with cellotriose must be occupied by a glucose unit. On the contrary, hydrolysis requires only the first two positive subsite to be occupied since cellobiose is the most abundant hydrolysis product. Several studies have shown the importance of the acceptor for various GHs (Pollock & Sharon, 1970 ▸; Armand *et al.*, 2001 ▸; Faijes *et al.*, 2003 ▸; Zakariassen *et al.*, 2011 ▸; Madhuprakash *et al.*, 2012 ▸; Wang *et al.*, 2014 ▸; Qin *et al.*, 2017 ▸; Garcia-Oliva *et al.*, 2019 ▸; Zhao *et al.*, 2021 ▸). To our knowledge, this structural study is the first to show how the acceptor length influences transglycosylation. The occupation of the +1 to +3 subsites brings the acceptor closer to Glu_A/B_ and the covalently linked donor, which could explain why transglycosylation is better with G3 as an acceptor than G2. Taken together with the data from our previous study on RBcel1, this work shows that occupation of the −3, −2 and +3 subsites induces definite movements of the key residues at the catalytic site (Glu135, Tyr201 and Glu245).

Finally, we have shown that the pH regulates the ratio of hydrolysis to transglycosylation. Indeed, transglycosylation products are more abundant at basic pH, where hydrolysis is reduced. With good leaving groups such as ClPNP, the GEI is formed without the protonic assistance of Glu135, which could remain deprotonated and poised for transglycosylation at basic pH. Whether this modulation of hydrolysis versus transglycosylation by pH is of biological significance remains to be investigated. RBcel1 was identified during a meta­genomic survey of an Antarctic soil sample collected from an oil-contaminated site (Berlemont *et al.*, 2009 ▸). Currently, its closest homolog is a GH5 enzyme from *P. saliphila* (locus WP_15030527), with 97% identity. Such homology suggests that RBcel1 originates from a *Pseudomonas* species that may be related to *P. saliphila* and *P. profundi*. Interestingly, both species show optimal growth at a basic pH of around 8.0–9.0 (Sun *et al.*, 2018 ▸; Zhang *et al.*, 2020 ▸), which would favor the transglycosylation activity of RBcel1.

The physiological role of bacterial transglycosylases remains to be elucidated. Berlemont *et al.* (2009 ▸) postulated that RBcel1 could be involved in cellulose synthesis. Indeed, RBcel1 has 48% identity to Ps_Cel5A from *P. stutzeri* strain A1501, a bacterium that forms a biofilm made of cellulose (Ude *et al.*, 2006 ▸). In some bacterial species such as *E. coli*, a gene encoding a GH8 endoglucanase, BcsZ, is commonly found in the *bcs* operon responsible for cellulose synthesis (Römling & Galperin, 2015 ▸). In *Rhizobium leguminosarum* and *Komatagaeibacter xylinus*, BcsZ has been shown to control the size and shape of cellulose fibrils (Robledo *et al.*, 2012 ▸; Nakai *et al.*, 2013 ▸). In the genome of *P. stutzeri*, however, there is no gene encoding a GH8 endoglucanase. Thus, Ps_Cel5A could achieve the same function as that of BcsZ (Berlemont *et al.*, 2009 ▸). However, one may wonder how transglycosylation could be relevant to the synthesis of bacterial cellulose. Several GHs acting as transglycosylases have been shown to be directly involved in the synthesis of plant cell-wall polysaccharides (Schröder *et al.*, 2004 ▸; Eklöf & Brumer, 2010 ▸; Nishikubo *et al.*, 2011 ▸; Franková & Fry, 2013 ▸). Their ability to ‘cut and paste’ allows the rearrangement of cell-wall polysaccharides during plant growth, cell-wall repair and cell differentiation (Franková & Fry, 2013 ▸). Such a re­arrangement activity is also found in transglycosylases from phytopathogenic fungi, as shown in *Botrytis cinerea* (Bi *et al.*, 2021 ▸). Since *P. stutzeri* is a plant commensal (Pham *et al.*, 2017 ▸; Sun *et al.*, 2021 ▸), an additional role of transglycosylating GHs could be in the host-interaction mechanism.

## Supplementary Material

PDB reference: RBcel1, E135Q mutant, 7p6g

PDB reference: E135Q mutant in complex with cellotriose, 7p6h

PDB reference: Y201F mutant, 7p6i

PDB reference: Y201F mutant, glycosyl-enzyme intermediate, 7p6j

Supplementary Table and Figures. DOI: 10.1107/S2059798321013541/jc5044sup1.pdf

## Figures and Tables

**Table 1:** Macromolecule-production information

	RBcel1_E135Q	RBcel1_Y201F
Source organism	Uncultured bacterium	Uncultured bacterium
Expression vector	pET-22b	pBAD-TOPO
Expression host	*Escherichia coli* BL21(DE3)	*Escherichia coli* MC1061
Complete amino-acid sequence of the construct produced	SVDLIGINVAGAEFTGGKLPGKHGTHYFFPPEGYFEYWSEQGIHTVRFPLKWERLQPSLNAELDDVYASLVDDMLDQAKENDIKVILDVHNYARYRKKVIGTEDVPVSAYQDLMERIAKRWQGHDALFAYDIMNQPYGSADKLWPAAAQAGIDGVRKYDKKRPLLIEGASWSSAARWPRYADELLKLKDPADNMVFSAHVYIDEDASGSYKKGPGKDFEPMIGVKRVEPFVNWLKEHGKKGHIGEGIPNDDERWLDAMDKLLAYLNENCIPINYWAAGPSWGNYKLSEPKDGEKRPQVALLKKYAAKDNCSDFGPAKAE	SVDLIGINVAGAEFTGGKLPGKHGTHYFFPPEGYFEYWSEQGIHTVRFPLKWERLQPSLNAELDDVYASLVDDMLDQAKENDIKVILDVHNYARYRKKVIGTEDVPVSAYQDLMERIAKRWQGHDALFAYDIMNEPYGSADKLWPAAAQAGIDGVRKYDKKRPLLIEGASWSSAARWPRYADELLKLKDPADNMVFSAHVFIDEDASGSYKKGPGKDFEPMIGVKRVEPFVNWLKEHGKKGHIGEGIPNDDERWLDAMDKLLAYLNENCIPINYWAAGPSWGNYKLSIEPKDGEKPQVALLKKYAAKDNCSDFGPAKAE

**Table 2:** Crystallization Values in parentheses are for the outer shell.

PDB code	7p6g	7p6h	7p6i	7p6j
Method	Vapor diffusion, hanging drop	Vapor diffusion, hanging drop	Vapor diffusion, hanging drop	Vapor diffusion, hanging drop
Plate type	EasyXtal (15-well)	EasyXtal (15-well)	EasyXtal (15-well)	EasyXtal (15-well)
Temperature (K)	293	293	293	293
Protein concentration (µ*M*)	385	385	400	400
Buffer composition of protein solution	20 m*M* sodium phosphate buffer pH 6.5	20 m*M* sodium phosphate buffer pH 6.5, 1 m*M* cellotriose	20 m*M* sodium phosphate buffer pH 6.5	20 m*M* sodium phosphate buffer pH 6.5, 1 m*M* cellotriose
Composition of reservoir solution	0.1 *M* Tris, 17.5% PEG 600 pH 8	0.1 *M* Tris, 17.5% PEG 600 pH 7.4	0.1 *M* Tris, 17.5% PEG 600 pH 7	0.1 *M* Tris, 20.5% PEG 600 pH 7
Volume and ratio of drop	1:1	1:1	1:1	1:1
Volume of reservoir (µl)	500	500	500	500

**Table 3:** Data collection and processing Values in parentheses are for the outer shell.

PDB code	7p6g	7p6h	7p6i	7p6j
Diffraction source	BM30A, ESRF	PROXIMA-2, SOLEIL	PROXIMA-2, SOLEIL	PROXIMA-2, SOLEIL
Wavelength (Å)	0.9798	0.9801	0.9801	0.9800
Temperature (K)	100	100	100	100
Detector	ADSC Quantum 315r CCD	ADSC Quantum 315r CCD	ADSC Quantum 315r CCD	Dectris EIGER X 9M
Crystal-to-detector distance (mm)	181.410	200.600	140.805	148.555
Rotation range per image (°)	0.33	0.5	0.5	0.1
Total rotation range (°)	182	180	360	360
Exposure time per image (s)	5.0	0.5	0.4	0.025
Space group	*P*2_1_2_1_2_1_	*P*2_1_2_1_2_1_	*P*2_1_2_1_2_1_	*P*2_1_
*a*, *b*, *c* (Å)	46.38, 99.52, 149.93	45.74, 99.79, 149.03	52.04, 63.06, 98.84	88.56, 90.54, 89.68
α, β, γ (°)	90, 90, 90	90, 90, 90	90, 90, 90	90, 118.77, 90
Mosaicity (°)	0.178	0.138	0.223	0.119
Resolution range (Å)	15.57–1.49 (1.53–1.49)	41.46–1.73 (1.77–1.73)	38.90–1.47 (1.51–1.47)	44.82–1.75 (1.76–1.75)
Total No. of reflections	800713	512215	800745	845927
No. of unique reflections	112952	71945	55794	124777
Completeness (%)	99.4 (93.1)	99.3 (93.0)	99.2 (90.7)	98.8 (93.3)
Multiplicity	7.090 (6.444)	7.120 (6.421)	13.935 (13.278)	6.780 (6.230)
〈*I*/σ(*I*)〉	22.27 (2.02)	14.59 (1.41)†	16.27 (1.72)†	13.54 (1.57)†
*R*_r.i.m._	0.046 (1.202)	0.088 (1.611)	0.113 (1.581)	0.073 (0.037)
Overall *B* factor from Wilson plot (Å^2^)	20.94	25.07	16.20	28.45

† The mean *I*/σ(*I*) in the outer shell is <2.0 but remains significant according to CC_1/2_ values (Karplus & Diederichs, 2015 ▸). The resolutions at which *I*/σ(*I*) falls below 2.0 are 1.76, 1.49 and 1.79 Å for PDB entries 7p6h, 7p6i and 7p6j, respectively.

**Table 4:** Structure solution and refinement

PDB code	7p6g	7p6h	7p6i	7p6j
Resolution range (Å)	15.57–1.49 (1.51–1.49)	41.46–1.73 (1.75–1.73)	38.90–1.47 (1.49–1.47)	44.82–1.75 (1.76–1.75)
Completeness (%)	99.4	99.3	99.1	99.0
σ Cutoff	*F* > 1.360σ(*F*)	*F* > 1.360σ(*F*)	*F* > 1.330σ(*F*)	*F* > 1.370σ(*F*)
No. of reflections, working set	107185 (3013)	68314 (2300)	51012 (1388)	118499 (3399)
No. of reflections, test set	5642 (159)	3595 (121)	4764 (92)	6235 (179)
Final *R*_cryst_	0.174 (0.2947)	0.167 (0.3701)	0.178 (0.4038)	0.172 (0.4221)
Final *R*_free_	0.193 (0.3323)	0.194 (0.3757)	0.193 (0.4633)	0.202 (0.4802)
Cruickshank DPI	0.075	0.148	0.082	0.206
No. of non-H atoms				
Protein	5108	5146	2570	10202
Ligand	20	84	24	302
Water	877	607	483	1159
Total	6005	5837	3077	11663
R.m.s. deviations				
Bond lengths (Å)	0.004	0.007	0.003	0.004
Angles (°)	0.673	0.879	0.650	0.728
Average *B* factors (Å^2^)				
Protein	26.98	30.10	19.33	36.86
Ligand	36.66	31.69	22.66	38.83
Water	38.65	40.84	34.09	41.91
Ramachandran plot				
Most favored (%)	98.42	98.11	97.81	98.03
Allowed (%)	1.58	1.89	2.19	1.90

**Table 5:** Apparent parameters for the hydrolysis of chromogenic cellooligo­saccharides by RBcel1 0.2 µ*M* enzyme was incubated with different substrates at concentrations ranging from 0.3 to 6 m*M*. The kinetic parameters were determined according to the release of ClPNP monitored by measuring the absorbance at 400 nm. The reactions were performed in 20 m*M* sodium phosphate pH 6.5 at 37°C. Standard error is given for each value with *n* = 3.

Substrate	Cellooligosaccharide equivalent	*k*_cat_ (s^−1^)	*K*_m_ (m*M*)	*k*_cat_/*K*_m_ (m*M* ^−1^ s^−1^)
PNP β-G1	G2	n.d.†	n.d.†	n.d.†
ClPNP β-G2	G3	n.d.†	n.d.†	n.d.†
ClPNP β-G3	G4	0.52 ± 0.02	2.37 ± 0.26	0.22 ± 0.02
ClPNP β-G4	G5	0.58 ± 0.01	0.30 ± 0.03	1.88 ± 0.19
ClPNP β-G5	G6	0.049 ± 0.01	0.34 ± 0.05	1.45 ± 0.14

† No significant activity was detected.
