# Supplementary material for: Highlighting the factors governing transglycosylation in the GH5_5 endo-1,4-β-glucanase RBcel1
Source: Acta Crystallogr D Struct Biol. 2022 Feb 18;78(Pt 3):278–89. doi: 10.1107/S2059798321013541 (PMC8900817; doi:10.1107/S2059798321013541)
Supplement: Supplementary file 1 [file d-78-00278-sup1.pdf]

Acta Cryst D STRUCTURAL BIOLOGY

Volume 78 (2022)

Supporting information for article:

Highlighting the factors governing transglycosylation in the GH5_5 endo-1,4-β-glucanase RBcel1

Laetitia Collet, Corinne Vander Wauven, Yamina Oudjama, Moreno Galleni and Raphaël Dutoit

Table S1 Real-space correlation coefficient (RSCC) and median B-factors of the ligands found in the structure of RBcel1_Y201 in complex with G3.

Subsites	RSCC / B-factors (Å^2)			
	Monomer A	Monomer B	Monomer C	Monomer D
-6	-	0.83 / 40	-	-
-5	-	0.76 / 44	0.86 /46	-
-4	0.91 / 30	0.89 / 40	0.91 / 37	0.80 / 43
-3	0.94 / 28	0.96 / 37	0.95 / 28	0.92 / 36
-2	0.96 / 24	0.90 / 34	0.94 / 25	0.96 / 31
-1	0.90 / 31	0.92 / 31	-	0.88 / 32
+1	0.82 / 42	0.87 / 39	0.94 / 24	0.84 / 39
+2	0.86 / 47	0.84 / 43	0.94 / 24	0.90 / 44
+3	-	-	0.82 / 46	-

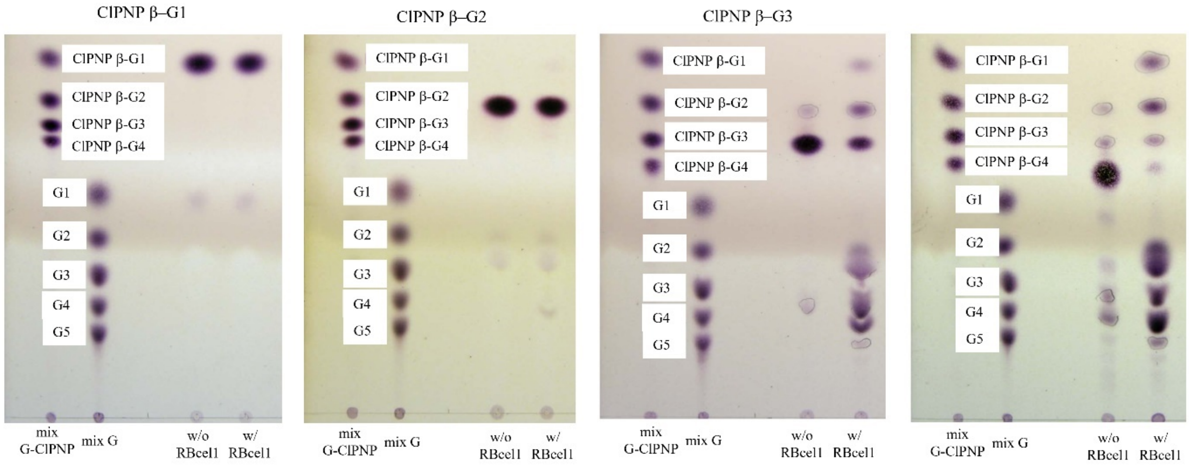

Figure S1 TLC analysis of reaction products from the activity of RBcel1_WT on ClPNP β-G1, ClPNP β-G2, ClPNP β-G3 and ClPNP β-G4. The first two tracks are reference ladders: the first mix (mix G-ClPNP) contains ClPNP β-G1 to G4 and the second mix (mix G) is made of G1, G2, G3, G4, and G5 (8 nanomoles of each). The tracks "w/ RBcel1" and "w/o RBcel1" correspond to the substrate incubated with and without RBcel1_WT, respectively.

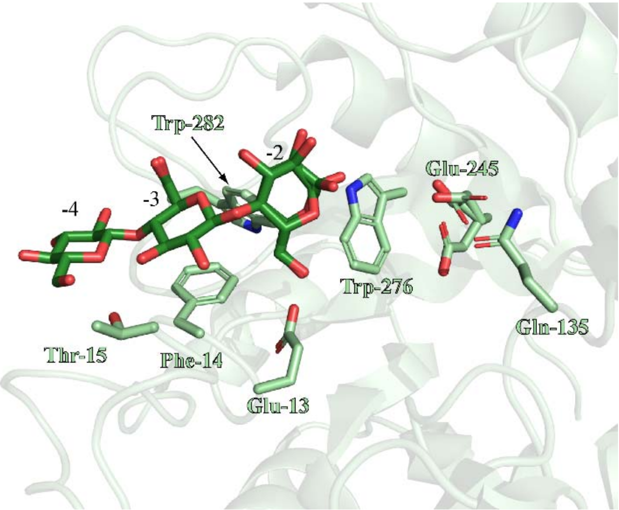

Figure S2 Close-up view of the -4 to -2 subsites of RBcel1_E135Q monomer B in complex with G3 (PDB code 7P6H). The residues composing the three subsites are emphasized as well as the two catalytic residues, Gln-135 and Glu-245.

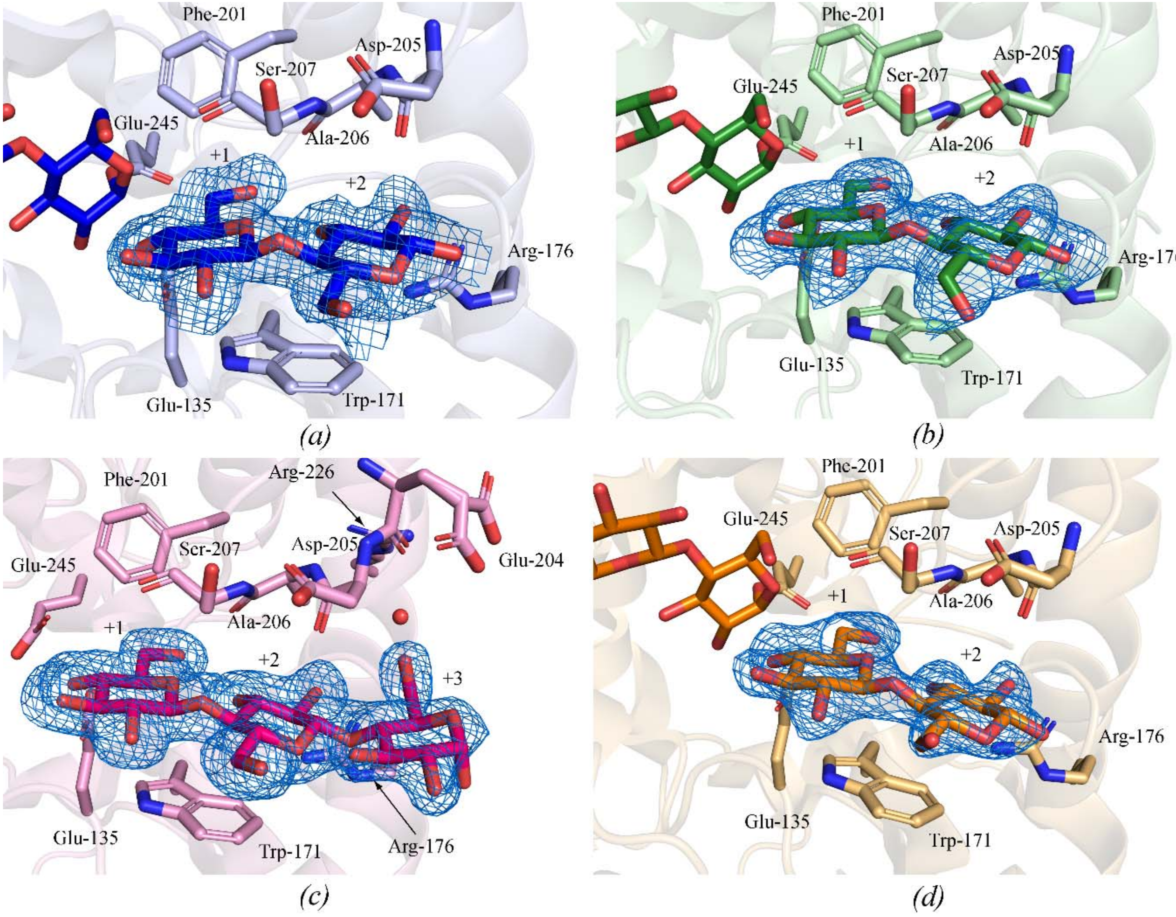

Figure S3 Close-up view of the positive subsites of RBcel1_Y201F in complex with G3. monomer B in complex with G3 (PDB code 7P6J). *(a)* Monomer A with G2 in its +1 to +2 subsites. *(b)* Monomer B with G2 in its +1 to +2 subsites. *(c)* Monomer C with G3 in its +1 to +3 subsites. *(d)* Monomer D with G2 in its +1 to +2 subsites. The water molecule interacting with glucose unit in the +3 subsite is

represented as a red sphere. The residues composing the three subsites are emphasized as well as the catalytic residues Glu-135 and Glu-245, and Phe-201. The $2F_o - F_c$ map around G3 is shown as a blue mesh.
